# Novel Insights into Redox-Based Mechanisms for Auranofin-Induced Rapid Cancer Cell Death

**DOI:** 10.3390/cancers14194864

**Published:** 2022-10-05

**Authors:** Elie Hatem, Nadine El Banna, Amélie Heneman-Masurel, Dorothée Baïlle, Laurence Vernis, Sylvie Riquier, Marie-Pierre Golinelli-Cohen, Olivier Guittet, Cindy Vallières, Jean-Michel Camadro, Xue Qiu, Niko Hildebrandt, Michel Lepoivre, Meng-Er Huang

**Affiliations:** 1 Institut Curie, PSL Research University, CNRS UMR 3348, Université Paris-Saclay, 91405 Orsay, France; 2 Université Paris-Saclay, CNRS UPR 2301, Institut de Chimie des Substances Naturelles, 91198 Gif-sur-Yvette, France; 3 Université Paris Cité, CNRS, Institut Jacques Monod, 75013 Paris, France; 4 NanoBioPhotonics (nanofret.com), Institute for Integrative Biology of the Cell (I2BC), Université Paris-Saclay, CNRS, CEA, 91400 Orsay, France; 5 Laboratoire Chimie Organique, Bioorganique, Réactivité et Analyse (COBRA), Université de Rouen Normandie, CNRS, INSA, 76821 Mont-Saint-Aignan, France

**Keywords:** auranofin, oxidative stress, redox regulation, cancer

## Abstract

**Simple Summary:**

One of the significant features of cancer cells is a persistent pro-oxidative status. Compared to their normal counterparts, the malignant cells are generally more dependent on antioxidants for cell survival and more vulnerable to further oxidative insults via pharmacological interventions. This is the biological basis of oxidative stress- or redox-based anticancer strategies. Auranofin (AUF) is a promising repositioning anticancer molecule with a multifaceted mode of action that could be cancer cell type- or dose-dependent. Using triple-negative breast cancer cells, we evidenced that thioredoxin reductase inhibition, the best-studied anticancer mechanism of AUF, may not be sufficient to induce efficient cell death. Cytotoxic doses of AUF elicited rapid and global intracellular oxidative stress. Based on the indications from redox proteome data, we showed experimentally that AUF treatment triggered a dose-dependent S-phase arrest and disintegration of the actin cytoskeleton structure. These findings on AUF-induced early effects should provide novel insights into the anticancer mechanisms of this promising molecule.

**Abstract:**

Auranofin (Ridaura^®^, AUF) is a gold complex originally approved as an antirheumatic agent that has emerged as a potential candidate for multiple repurposed therapies. The best-studied anticancer mechanism of AUF is the inhibition of thioredoxin reductase (TrxR). However, a number of reports indicate a more complex and multifaceted mode of action for AUF that could be cancer cell type- and dose-dependent. In this study, we observed that AUF displayed variable cytotoxicity in five triple-negative breast cancer cell lines. Using representative MDA-MB-231 cells treated with moderate and cytotoxic doses of AUF, we evidenced that an AUF-mediated TrxR inhibition alone may not be sufficient to induce cell death. Cytotoxic doses of AUF elicited rapid and drastic intracellular oxidative stress affecting the mitochondria, cytoplasm and nucleus. A “redoxome” proteomics investigation revealed that a short treatment with a cytotoxic dose AUF altered the redox state of a number of cysteines-containing proteins, pointing out that the cell proliferation/cell division/cell cycle and cell–cell adhesion/cytoskeleton structure were the mostly affected pathways. Experimentally, AUF treatment triggered a dose-dependent S-phase arrest and a rapid disintegration of the actin cytoskeleton structure. Our study shows a new spectrum of AUF-induced early effects and should provide novel insights into the complex redox-based mechanisms of this promising anticancer molecule.

## 1. Introduction

One of the significant features of cancer cells is a persistent pro-oxidative state. Several carcinogenesis- and cancer progression-linked events, such as activation of oncogenes, aberrant metabolism and mitochondrial dysfunction, can increase intracellular reactive oxygen species (ROS) levels, leading to intrinsic oxidative stress. Therefore, cancer cells are generally addicted to antioxidants for cell survival and more vulnerable to further oxidative insults via pharmacological interventions targeting cellular redox systems. Thus, the difference in intrinsic ROS levels and redox status between normal and cancer cells provides a potential window to develop redox-based therapeutic strategies [1,2]. Among the promising oxidative stress-based anticancer molecule, auranofin (Ridaura^®^, AUF) has received much attention. AUF is an orally administered, gold (Au)-containing old drug approved by the FDA in 1985 for treatment of rheumatoid arthritis [3]. Interestingly, AUF has emerged as a candidate for multiple repurposed therapies including microbial infections and cancers [4,5]. The anticancer activity of AUF has been observed in a variety of human cancer cell lines and tumor models [6]. These studies have led to the clinical trials of AUF in different types of cancers (ClinicalTrials.gov—www.clinicaltrials.org, accessed on 10 September 2022).

The anticancer mechanism of AUF is not fully understood. The best-studied mechanism is the inhibition of thioredoxin reductase (TrxR) activity through AUF’s high affinity for selenol-containing residues of the active site of both cytoplasmic TrxR1 and mitochondrial TrxR2, resulting in modifications of the cellular redox state, over-production of ROS, oxidative damage and apoptosis [4,7,8]. On the other hand, as for several other gold compounds [9], it was demonstrated that AUF targets the proteasome [10,11]. AUF inhibits 19S proteasome-associated deubiquitinases (DUBs) but does not inhibit the three main catalytic peptidase activities of 20S proteasome, thereby inhibiting the degradation of ubiquitinated proteins, consequently inducing cell apoptosis.

Earlier studies suggested that AUF non-selectively inhibits DNA, RNA and protein synthesis in human HeLa cells and murine B16 cells at cytotoxic concentrations [12,13]. However, a rapid cellular response to the AUF toxicity, unlike the more delayed response observed for several anticancer agents, such as adriamycin and actinomycin D, led some authors to suggest that the action of AUF is due to its effects on cellular processes other than on DNA, RNA or protein synthesis [14]. AUF also inhibits several cancer signaling pathways, contributing to its antiproliferative effects. For example, AUF inhibits STAT3 and telomerase activity in MDA-MB-231 breast cancer cells [15] and induces the apoptosis of multiple myeloma cells through both down-regulation of STAT3 and inhibition of NF-κB activity [16]. AUF also activates the FOXO3 tumor suppressor [17] and inhibits the protein kinase Cι (PKCι) signaling in ovarian cancer models [18] as well as the PI3K/AKT/mTOR axis in non-small cell lung cancer cells [19].

Overall, these numerous studies underline the effective anticancer activity of AUF but point to a complex and multifaceted mode of action that could be partially cancer cell type- or dose-dependent. Another interesting aspect is its potential synergy in combination with other anticancer drugs [6]. Recently, we identified that the combination of AUF and vitamin C efficiently kills triple-negative breast cancer (TNBC) cells [20]. In the perspective of a redox-based anticancer strategy, AUF is a particularly promising redox-modulating molecule that warrants investigating further its mechanisms of action. In this study, we intended to investigate and identify the early events that explain AUF anticancer activity.

## 2. Materials and Methods

### 2.1. Cell Line and Reagents

The TNBC cell line MDA-MB-231 was purchased from American Type Culture Collection. Cells were grown in DMEM (Eurobio) supplemented with 10% FBS, 2 mM L-glutamine, 1x non-essential amino acids and 100 U/mL penicillin and 100 μg/mL streptomycin. Four other TNBC cell lines, including MDA-MB-436, HCC-1937, MDA-MB-468 and BT-549 were kindly provided by Dr Fabien Reyal and Dr Alice Pinherio in the Translational Research Department of Institut Curie. The cell line was authenticated using a standard DNA microsatellite short tandem repeat (STR) method. Cells were incubated in humidified atmosphere at 37 °C with 5% CO_2_ in air.

AUF was purchased from Enzo Life Sciences. N-acetyl-L-cysteine (NAC), DMSO, H_2_O_2_ were purchased from Sigma-Aldrich. 3-(4,5-dimethylthiazol-2-yl)-2,5-diphenyltetrazolium bromide (MTT), 5-bromo-2′-deoxy-uridine (BrdU), propidium iodide (PI), anti-GFP antibody and anti-GADPH antibody were from Thermo Fisher Scientific. The anti-PRDX3 was from Abcam, the anti-ß-actin antibody was from Santa Cruz Biotechnology, and the FITC mouse anti-BrdU antibody was from BD Biosciences.

### 2.2. Evaluation of Cytotoxicity

Cytotoxicity was mainly assessed using the MTT assay. For 24 h AUF treatment condition, 1.25 × 10^4^ cells/well were seeded in 96-well plates and cultured for 24 h prior to AUF treatments. A longer treatment time requires lower cell density at the starting point to avoid over-confluence. Thus, 6 × 10^3^ cells/well and 3 × 10^3^ cells/well were used, respectively, for 48 h and 72 h treatments. For colony formation assay, 3.5×10^5^ cells/well were seeded in 6-well plates for 24 h and were subjected to defined treatments for another 24 h. Cells were then harvested, washed, plated at various densities in 6-well plates and cultured for 14 days. Colonies were stained with 0.5% crystal violet solution and scanned with Odyssey Imager (Li-COR Biosciences). For flow cytometry-based cell death assay, cells seeded in 6-well plates as above were treated with defined conditions, harvested, and washed in PBS containing 1% FBS. After PI (1 µg/mL) staining; then, cells were analyzed by a Cytoflex flow cytometer (Beckman Coulter, Brea, CA, USA).

### 2.3. Thioredoxin Reductase Assay

Cells were seeded in 10 cm dishes at a density of 2 × 10^5^ cells/mL (10 mL volume of medium) for 24 h and subjected to defined treatments. Cells were washed, harvested and lysed using M-PER Mammalian Protein Extraction Reagent (Thermo Fisher Scientific, Waltham, MA, USA). Protein concentrations were determined by the Pierce BCA Protein Assay Kit, and an equal amount of proteins was used for each condition. The TrxR activity was determined with a Thioredoxin Reductase Assay Kit (Sigma-Aldrich, St. Louis, MO, USA) according to the manufacturer’s protocol using a reaction scheme for a 96-well plate assay. The reduction of 5,50-dithiobis-(2-nitrobenzoic acid) (DTNB) was followed by measuring optical density (OD) at 412 nm using an enzymatic kinetic program with a delay of 5 min and 20 measurements during 20 min. Reactions were carried out at 25 °C. Since in the crude biological sample, other enzymes, such as glutathione reductase and glutathione peroxidase, could also reduce DTNB, the DTNB reduction by the sample in the presence of the TrxR specific inhibitor provided in the kit was used to determine TrxR-specific activity. The difference between the two reads is the DTNB reduction by TrxR for a given sample. TrxR activity is reported and plotted with the first reading at 412 nm for each sample set to be 0.

### 2.4. Oxidative Stress Assessment

For the detection of reduced and oxidized peroxiredoxin 3 (PRDX3), 3.5 × 10^5^ cells/well were seeded in 6-well plates for 24 h before subjecting them to different treatments. Redox Western blots were processed as described previously [21]. For HyPer sensors, mammalian expression vectors encoding HyPer targeted to the nucleus (nuc-HyPer) with a nuclear localization signal were purchased from Evrogen. HyPer targeted to the cytosol (cyto-HyPer) with a Nuclear Export Signal (NES) was constructed in our laboratory. The PCR product, fusing NES in frame to the 3′ end of HyPer, was then cloned in a pCMV/myc/cyto vector (Thermo Fisher Scientific). HyPer-expressing vectors were transformed in MDA-MB-231 cells using jetPRIME reagents (Polyplus Transfection). AUF treatment experiments were carried out 24 h after transfection. Protein extraction and analysis of oxidized and reduced forms of HyPer sensors by redox Western blots were performed according to our previously reported method [21]. HyPer fluorescent imaging in living cells was acquired with a Nikon inverted spinning-disk microscope using excitation laser lines 405 nm and 491 nm and an emission range set from 500 to 530 nm according to the method described by Mishina et al. [22]. Time-lapse imaging was recorded every 1 min using multi-channel 4D (x,y,z,t). Images were exported to the ImageJ software, and the ratio (491/405 nm) was computed by dividing the 491 nm by the 405 nm image pixel by pixel. The final 491/405 nm ratio images after Z projection used the ImageJ look-up table ‘Fire’ for creating false color ratio images.

### 2.5. Quantitative Redoxome Analysis

MDA-MB-231 cells were seeded in 6-well plates at a density of 3.5 × 10^5^ cells/well (2 mL volume of medium) and allowed to attach for 24 h before being subjected to defined treatments. Proteins containing oxidized thiols were extracted according to our previously published protocol [23]. Digests of oxidized proteins were analyzed in triplicate with an Orbitrap Fusion Tribrid equipped with an EASY-Spray ion source. Label-free liquid-chromatography tandem mass spectrometry (LC-MS/MS) acquisition was performed. Peptides from MS/MS data were processed with Proteome Discoverer Software (v 2.1) using the Sequest search node. All results were 1% FDR filtered before exporting. The resulting files were imported into Progenesis-Qi software for report edition. Variations of protein abundance were considered as validated if their Anova *p* values were < 0.05. The PANTHER classification system (http://www.pantherdb.org, accessed on 10 September 2022) [24] and DAVID software (https://david.ncifcrf.gov, version 6.8, accessed on 10 September 2022) [25] were used to determine functional protein classes and biological processes enriched from Gene Ontology (GO) database. Functional annotations with *p* < 0.05 (EASE score) (modified Fisher’s exact test) were selected. The proteomics data have been deposited to the ProteomeXchange Consortium via the PRIDE [26] partner repository with the dataset identifier PXD036558.

### 2.6. BrdU Incorporation and Cell Cycle Analysis

MDA-MB-231 cells were seeded in 6-well plates (3.5 × 10^5^ cells/well) for 24 h and were subjected to treatments with various concentrations of AUF for 1 h. Cells were then washed and released in fresh medium. At defined time points of post-treatment recovery (0, 2, 6, 24 h), these cells were labeled with 10 µM BrdU for 1 h. Labeled cells were washed by PBS, detached by trypsin treatment, collected and fixed in cold 70% ethanol. The fixed cells were washed in wash solution (50 mM Tris/HCl, pH 7.4, 150 mM NaCl, 0.5% BSA) and incubated in 2 M HCl for 20 min at room temperature. After neutralizing acidity with 0.1 M sodium borate buffer (pH 8.5) for 5 min, cells were washed, resuspended and incubated for 1 h at room temperature in a solution of FITC anti-BrdU antibody diluted in 50 mM Tris/HCl, pH 7.4, 150 mM NaCl, 0.5% Tween 20 and 0.5% BSA. Finally, cells were washed in wash solution, resuspended and incubated in wash solution containing 10 µg/mL of PI and 0.5 mg/mL of RNAse A for 30 min at room temperature. Samples were analyzed using a Cytoflex flow cytometer (Beckman Coulter), and data were analyzed using CyExpert software. For experiments with a reverse scheme in which MDA-MB-231 cells were first labeled with 10 µM BrdU for 1 h, and then were left untreated or exposed to AUF 6 µM for 30 min, analyses were performed at 0, 4, 8 and 24 h during the post-treatment recovery. Cell collection, fixation, DNA hydrolysis, FITC anti-BrdU antibody and PI staining were proceeded as above. Samples were analyzed using a FACSCanto flow cytometer (Becton Dickinson) and data were analyzed using Flowjo software.

### 2.7. Quantitation of Cellular dTTP and dGTP

dTTP and dGTP levels in MDA-MB-231 cells were measured by a recently developed rolling circle amplification (RCA)- and Förster resonance energy transfer (FRET)-based dNTP detection assay [27]. Briefly, 5 × 10^6^ cells were seeded in 15 cm dishes. After 24 h, they were treated with 1 or 6 µM AUF for 30 min or 2 h. Cells were then harvested by centrifugation and extracted with ice-cold 60% methanol (5 × 10^6^ cells/mL), boiled for 5 min and centrifuged at 17,000× *g* for 30 min. The supernatant was collected and lyophilized, and it was resuspended with nuclease-free water at a concentration corresponding to 1 × 10^6^ cells/30 µL that was used for RCA-FRET dTTP and dGTP quantification assays.

### 2.8. Actin Filament Imaging

Cells grown on coverslips and submitted to different treatments were rinsed with PHEM buffer (60 mM PIPES, 25 mM HEPES pH 6.9, 5 mM EGTA and 2 mM Mg acetate), pre-lysed in PHEM buffer containing 0.5% Triton X-100 and fixed in PHEM buffer containing 3.7% paraformaldehyde/0.02% glutaraldehyde. Autofluorescence of paraformaldehyde was diminished by incubation of cells in NH_4_Cl 50 mM/PBS pH 7.4 for 10 min. Cells were permeabilized with 0.1% Triton X-100 (1% BSA/PBS) for 5 min at room temperature and rinsed with PBS. To localize actin filaments, cells were incubated with rhodamine–phalloidin (Invitrogen) for 20 min at room temperature in a humid chamber at concentrations according to the manufacturer’s protocol. The coverslips were rinsed in PBS and mounted on the slide using Prolong Gold Antifade Mountant (Invitrogen). Images were obtained using a Leica SP8 upright confocal microscope. Z-stacks (about 15 images taken at different z-planes) encompassing the entire volume of the cells were taken. The merged z-stacks were obtained using the ImageJ software. Actin filament intensity per cell area was determined indirectly by measuring the intensity of fluorescence (phalloidin staining) using the ImageJ software, and more than 30 cells per sample were measured.

### 2.9. Statistical Analysis

Data were presented as the mean ± SD. Wherever necessary, an unpaired t test with Welch’s correction available in the GraphPad Prism 7 software was performed to compare the difference between differently treated cells. *p* < 0.05 is considered statistically significant.

## 3. Results

### 3.1. AUF-Induced Cytotoxicity in TNBC Cells

**Figure 1:**
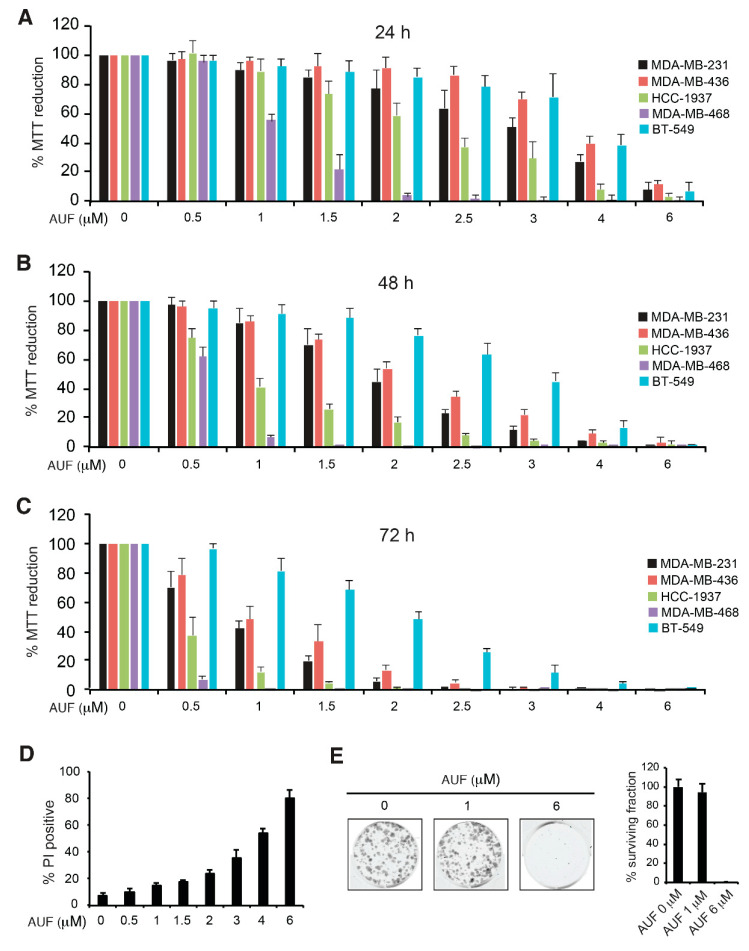
Cytotoxicity following AUF treatments. (**A**–**C**) Five TNBC cell lines were treated with AUF at indicated concentrations for 24 h (**A**), 48 h (**B**) and 72 h (**C**), and cytotoxicity was measured with the MTT assay. Bar graphs show the mean ± SD of at least three independent experiments. The percentage of MTT reduction was calculated relative to AUF non-treated cells (set as 100%). (**D**) MDA-MB-231 cells were treated with AUF at indicated concentrations for 24 h and stained with PI before proceeding to the cytometric analysis. Bar graphs show mean ± SD of at least three independent experiments. (**E**) Colony formation of MDA-MB-231 cells following treatment with AUF 1 μM and 6 μM for 24 h. The percentage of surviving fraction was calculated relative to non-treated cells.

TNBC represents a heterogeneous and aggressive breast cancer subtype with a poor prognosis. Five TNBC cell lines, MDA-MB-231, MDA-MB-436, HCC-1937, MDA-MB-468, and BT-549, were exposed to increasing concentrations of AUF ranging from 0.5 to 6 µM. To avoid over-confluence in culture in the course of the experiments, we seeded 1.25 × 10^4^ cells/well of 96-well plate for 24 h treatment, 6 × 10^3^ cells/well for 48 h treatment and 3 × 10^3^ cells/well for 72 h treatment. As expected, lower cellular density and longer treatment duration reduced the IC50 values for each cell line (Figure 1A–C). Globally, AUF displayed dose-dependent cytotoxicity to all five TNBC cells, MDA-MB-468 cells being highly sensitive and BT-549 cells being more resistant. MDA-MB-231, the most frequently used TNBC cell line, showed an intermediate sensitivity and therefore was used in further studies. The IC50 of MDA-MB-231, estimated by MTT assay under condition of 1.25 × 10^4^ cells/well for 24 h treatment was about 3 µM. PI, which is membrane-impermeant and is frequently used to identify dead cells, confirmed a dose-dependent decrease in cell viability after 24 h treatment (Figure 1D). Treatment with 1 µM AUF had little impact on colony formation, while 6 µM led to a complete loss of ability to form colonies (Figure 1E). To evaluate the early impact of AUF in the following studies, we usually employed AUF under 3 µM as moderate concentrations, and 6 µM AUF, which is about twice the IC50 and can induce a large proportion of cell death as a cytotoxic condition.

### 3.2. AUF-Mediated TrxR Inhibition Alone May Not Be Sufficient to Induce Cell Death

**Figure 2:**
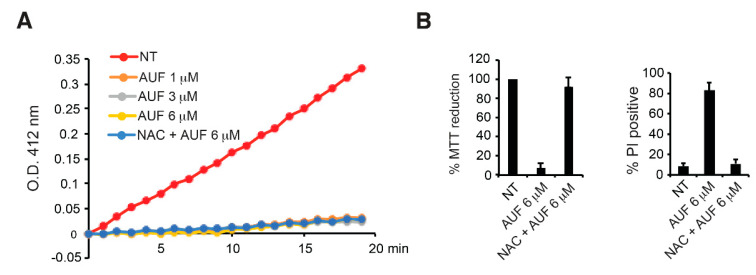
Inhibition of TrxR activity following AUF treatments. (**A**) Total TrxR activity of the cells with indicated treatments was measured, and one out of three independent experiments is presented. The time course of DTNB reduction was monitored at 412 nm over 20 min. OD at 412 nm of the first reading for each sample is set to be 0. (**B**) MDA-MB-231 cells were treated with 6 µM AUF alone or were pre-treated with 2 mM NAC for 1 h before adding 6 µM AUF. Cytotoxicity was measured with the MTT assay (left panel) and PI assay (right panel). Bar graphs show means ± SD of three independent experiments.

The AUF-mediated inhibition of TrxR activities and consequent modifications of the cellular redox state and oxidative damage are often proposed as one of central mechanisms for AUF anticancer activity. To verify the link between AUF-induced cell death and AUF-mediated TrxR inhibition, TrxR activity was monitored in MDA-MB-231 cells exposed to various concentrations of AUF. Cell extracts prepared from MDA-MB-231 cells treated with 1, 3 and 6 µM of AUF for 4 h showed a drastic inhibition of the reduction of DTNB mediated by TrxR (Figure 2A). Interestingly, 1 µM of AUF, which only has a low cytotoxic effect on the cells (as revealed by the MTT, PI and clonogenic assays) (Figure 1A,D,E), conferred a similar inhibitory effect on TrxR activity as 3 and 6 µM, suggesting that TrxR inhibition alone may not be directly correlated to AUF cytotoxicity.

NAC has been widely used as an antioxidant or a ROS scavenger. NAC is able to suppress AUF-induced cell death and restore cellular viability (Figure 2B). It is also reported that NAC, by changing the chemical structure of AUF in PBS, completely reverses AUF-induced proteasome inhibition and apoptosis in HepG2 and MCF-7 cells. Therefore, the effect of NAC could be simply attributed to AUF inactivation in the medium [10]. To check whether the presence of NAC rescues TrxR activity in AUF-treated cells, cells were pre-treated with 2 mM NAC for 1 h before adding 6 µM AUF in the medium. Under this condition that suppressed cytotoxicity (Figure 2B), inhibition of the reduction of DTNB in the cells was maintained (Figure 2A), which indicates that TrxR activity was still largely inhibited by 6 µM AUF. Taken together, our observations confirm that AUF is an efficient TrxR inhibitor, whereas such an inhibition of TrxR activity alone may not be sufficient to induce efficiently cell death. The experiment with NAC also suggests that the protective effect of NAC may not be simply due to the deactivation of AUF by NAC in culture medium, although interaction between AUF and NAC may exist under some conditions (it may be dependent on NAC concentrations).

### 3.3. AUF Treatment Induces Rapid and General Intracellular Oxidation

**Figure 3:**
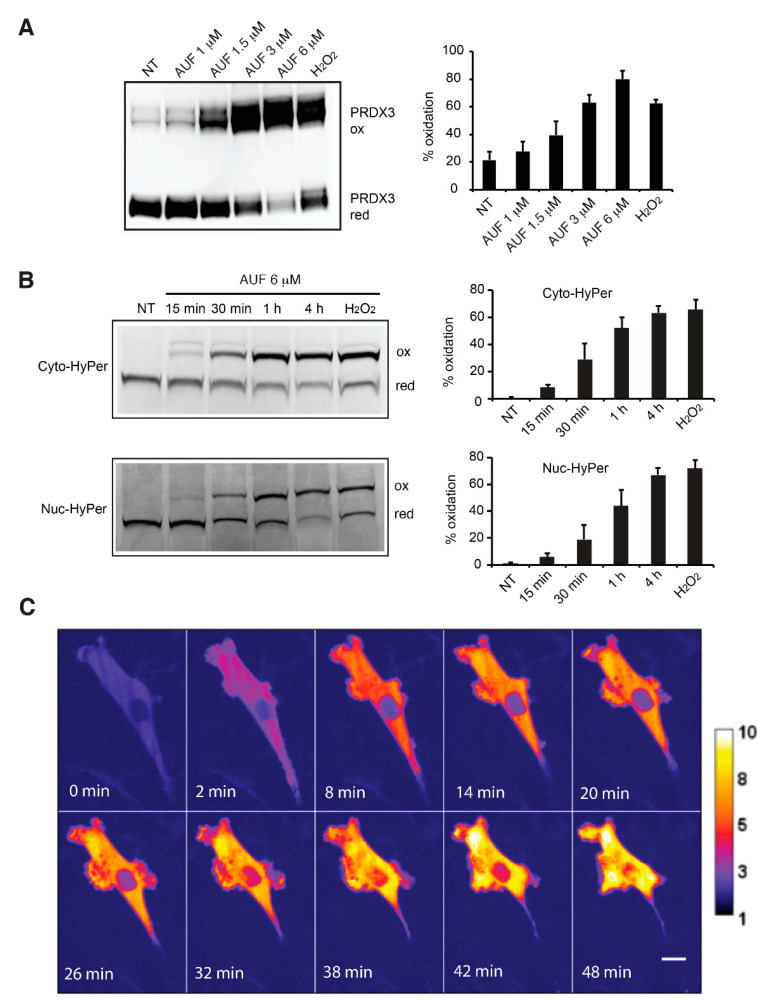
AUF-induced intracellular oxidative stress. (**A**) Redox state of PRDX3 was assessed using redox Western blot in non-treated (NT) and cells treated with indicated concentrations of AUF for 30 min. Treatment with 100 μM H_2_O_2_ for 30 min was used as a positive control. ox, oxidized form; red, reduced form. A representative image is presented. Graphs show the quantification of oxidized (%) versus total PRDX3 protein. Bar graphs show mean ± SD of three independent experiments. (**B**) Redox state of cyto- and nuc-HyPer sensors in MDA-MB-231 cells treated with 6 μM AUF for indicated time. Treatment with 100 µM H_2_O_2_ for 30 min was used as a positive control. Graphs show the quantification of oxidized (%) versus total HyPer protein. ox, oxidized form; red, reduced form. All bar graphs show mean ± SD of three independent experiments. (**C**) Representative confocal live cell images of a MDA-MB-231 cell expressing cyto-HyPer. AUF (6 μM) was added between the first and second frames shown and time of treatment is indicated. Stacks of images were taken with excitation at 405 and 491 nm, respectively. Maximum projections of these stacks were used for the calculation of the ratio images. The color scale for the ratio values indicates maximal reduced HyPer in blue and maximal oxidized HyPer in yellow. Scale bar = 10 µm. Original blots/gels can be found at supplementary figures.

Intracellular ROS accumulation following AUF treatment has been observed in a number of studies [4]. We also previously observed that 6 µM AUF treatment appears to induce more severe oxidative stress in mitochondria than in cytoplasm [20]. To refine the evaluation of the redox alteration induced by AUF, the oxidation of mitochondria-localized PRDX3 was monitored in MDA-MB-231 cells treated by several concentrations of AUF. An increase in oxidized PRDX3 dimer was detectable upon treatment with 1.5 µM AUF for 30 min, while a predominant shift from reduced PRDX3 monomer to oxidized dimer was obtained after treatment with 3 and 6 µM for 30 min (Figure 3A and Supplementary Figure S1). We further analyzed dynamic cytoplasmic and nuclear H_2_O_2_ accumulation using H_2_O_2_-specific HyPer sensors transiently expressed in MDA-MB-231 cells. The oxidation status of cytoplasm-targeted cyto-HyPer and nucleus-targeted nuc-HyPer were monitored by a redox Western blot at different time points following the addition of 6 µM AUF to the cell culture (Figure 3B and Supplementary Figure S2). Prior to AUF treatment, HyPer sensors were in a highly reduced state. The oxidation of cyto- and nuc-HyPer was clearly visible after 30 min and continued to progress up to 4 h. To further refine the rapidity of HyPer oxidation, we followed the oxidation of cyto-HyPer in live MDA-MB-231 cells by confocal microscopy. The cells displayed a rapid and significant increase in fluorescence intensity, which was detectable 2 min after the addition of 6 µM AUF (Figure 3C). Although the fluorescence emission of HyPer sensor is pH-sensitive [28], it is highly unlikely that such a short exposure to AUF induced significant pH modification. It is of note that redox Western blot detection of oxidized and reduced form of HyPer is not pH-sensitive. Taken all together, the data indicate a rapid, massive and sustained intracellular oxidative stress as a very early event following the addition of a cytotoxic concentration of AUF.

### 3.4. Redox Proteome Analysis Reveals a Large Spectrum of AUF-Induced Effects

Because a cytotoxic concentration of AUF elicited a rapid intracellular oxidative stress as described above, we decided to perform MS-based redox proteomics analysis to evaluate the oxidation of the cysteine (Cys) proteome in response to AUF treatment. Quantitative proteomic comparison was performed between 6 µM AUF treated and non-treated cells in two independent biological experiments, each with three technical replicates. We set criteria to include peptidyl Cys detected in both experiments with at least one of two values greater than 1.5 (50% more or less oxidized than non-treated cells, in the same direction of change) and with an ANOVA *p* value < 0.05 for each experiment. For this, the proteins that exhibited an increased or decreased oxidation with an ANOVA *p* value < 0.05 compared with the basal redoxome of non-treated cells were retained. There were 632 proteins displaying a change in their oxidation profile under treatment for one biological experiment and 723 proteins for the other. Comparison between the two protein lists using a Venn diagram revealed 161 common proteins according to our criteria (Supplementary Table S1). Among them, 113 were more oxidized, and 48 were less oxidized following AUF treatment. A set of antioxidant proteins such as thioredoxin 1 (TRX1), glutathione synthetase (GSS) and glutathione S-transferase Mu 3 (GSTM3) were among the significantly oxidized proteins, confirming the validity of the employed redoxome technique.

**Figure 4:**
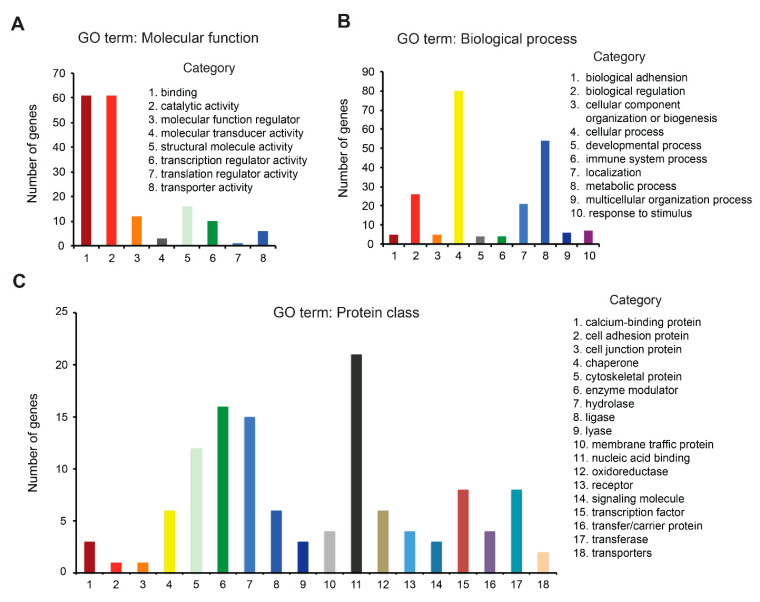
Analysis of the 161 proteins which exhibited an increased or decreased oxidation over 1.5-fold with a *p* value < 0.05 following 6 µM AUF treatment for 30 min compared with the basal redoxome of non-treated cells using the PANTHER classification system. These proteins were classified in terms of their Molecular function (**A**), Biological process (**B**) and Protein class (**C**).

A first step for functional interpretation of the resultant 161 proteins was to classify them based on GO term, such as *molecular function*, *biological process* and *protein class* using the PANTHER classification system. According to *molecular function*, most of the proteins belonged to the subcategories of *binding* and *catalytic activity* (Figure 4A), whereas most *biological process* proteins belonged to the subcategories of *cellular process*, *metabolic process*, *cellular component organization* or *biogenesis* (Figure 4B). According to protein class, most of the proteins belonged to the subcategory of *nucleic acid binding* (Figure 4C), including several ribosomal proteins and DNA helicases such as 60S ribosomal protein L11 (RPL11), 40S ribosomal protein S5, ATP-dependent DNA helicase Q1 (RECQL), and DNA replication licensing factor MCM6. These 161 proteins with significant fold change were also subjected to analysis by DAVID software to explore the biological process involved. The results indicated that 50 GO-biological process terms were significantly enriched (*p* values < 0.05), revealing a large spectrum of AUF-induced effects. The top 10 biological process terms are listed in Table 1. Globally, the results of enrichment analysis and nature of proteins identified seems to point to cell proliferation/cell division/cell cycle and cell–cell adhesion/cytoskeleton as the most affected pathways. However, it is essential to keep in mind that the number of proteins affected in a given process following AUF treatment does not necessarily correlate to the origin of AUF-induced cytotoxicity. Nevertheless, the redox proteome analysis guided us to perform experimental verification of the two pathways being the most affected quantitatively.

### 3.5. AUF Treatment Induces a Dose-Dependent Inhibition of DNA Replication

**Figure 5:**
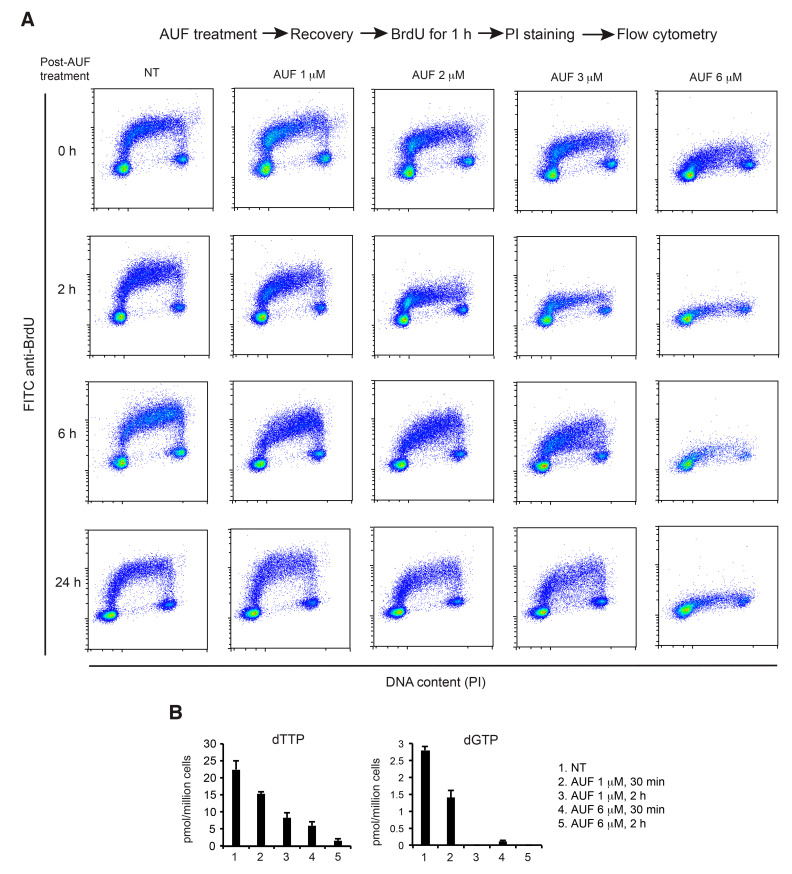
Effect of AUF on BrdU incorporation and on intracellular dTTP and dGTP levels. (**A**) MDA-MB-231 cells, non-treated (NT) or treated with 1, 2, 3, or 6 µM AUF for 1 h, were released in fresh medium. At 4 time points of post-treatment recovery (0, 2, 6, 24 h), these cells were labeled with BrdU for 1 h followed by PI staining and flow cytometry analysis. Representative graphs of three experiments are shown. (**B**) Intracellular dTTP and dGTP in MDA-MB-231 cells treated under indicated conditions. Bar graphs show mean ± SD of two independent experiments.

Asynchronous MDA-MB-231 cells were exposed to 0, 1, 2, 3 and 6 µM of AUF for 1 h. At defined time points of post-treatment recovery (0, 2, 6 and 24 h), these cells were incubated with BrdU for 1 h. BrdU, a thymidine analogue, can be incorporated into actively synthesizing DNA and revealed by FITC anti-BrdU antibody. As shown in Figure 5A, in non-treated cells, BrdU added to the culture medium was incorporated into DNA during DNA replication. BrdU content was observed in S-phase, forming a typical horse shoe-shaped arc from G1 in the lower left up to S-phase and down into the lower right for G2/M. Samples treated with lower concentrations of AUF (1, 2, 3 µM) revealed a dose-dependent decrease in BrdU positive events in the S-phase at early time points of post-treatment recovery (0 h and 2 h), indicating a rapid but transient or partial inhibition of BrdU incorporation (Figure 5A). In contrast, upon exposure to AUF 6 µM, BrdU incorporation was rapidly stopped and remained totally inhibited for at least 24 h.

We then further confirmed the effect of 6 µM AUF on DNA replication using a reverse scheme in which asynchronous MDA-MB-231 cells were first labeled with BrdU for 1 h, and then were left untreated or exposed to AUF 6 µM for 30 min. Analyses were performed at 0, 4, 8 and 24 h during the post-treatment recovery. As shown in Supplementary Figure S3, in non-treated cells, BrdU added to culture medium was incorporated into DNA during DNA replication. In the cell population sampled at 24 h, a complete cell cycle was obtained. In contrast, treatment of 6 µM AUF for 30 min blocked the progression of cells in the S-phase for at least 24 h. Taken together, these results demonstrate that DNA replication is rapidly impaired after AUF treatment. Lower concentrations of AUF result in transient delay of DNA replication, while higher cytotoxic concentrations of AUF may abolish DNA replication in an irreversible manner, which could link to AUF-induced cytotoxicity.

Efficient DNA replication and repair depend largely on ribonucleotide reductase (RNR) activity which, in turn, depends on thioredoxins and glutaredoxins as electron donors. Nicotinamide adenine dinucleotide phosphate (NADPH), the ultimate electron donor, reduces thioredoxins and glutaredoxins via TrxR or glutathione reductase and glutathione, respectively. AUF is an inhibitor of TrxR and affects also the glutathione pathway [20]. Therefore, we suspected that AUF treatment impacts RNR activity and dNTP synthesis. We quantified dTTP and dGTP levels in MDA-MB-231 cells treated with 1 µM AUF (concentration inhibiting TrxR activity) and 6 µM AUF (cytotoxic concentration) for 30 min and 2 h. We found that dTTP and dGTP levels experienced significant decreases in an AUF-dose and treatment time-dependent way (Figure 5B). The depletion of dNTP pools provokes DNA replication arrest by several mechanisms. dNTPs decrease might thus be responsible for DNA replication arrest after AUF treatment, leading to persistent replication fork stalling and ultimately promoting DNA strand breaks and cell death.

### 3.6. AUF Treatment Induces a Rapid Disintegration of the Actin Cytoskeleton Structure

**Figure 6:**
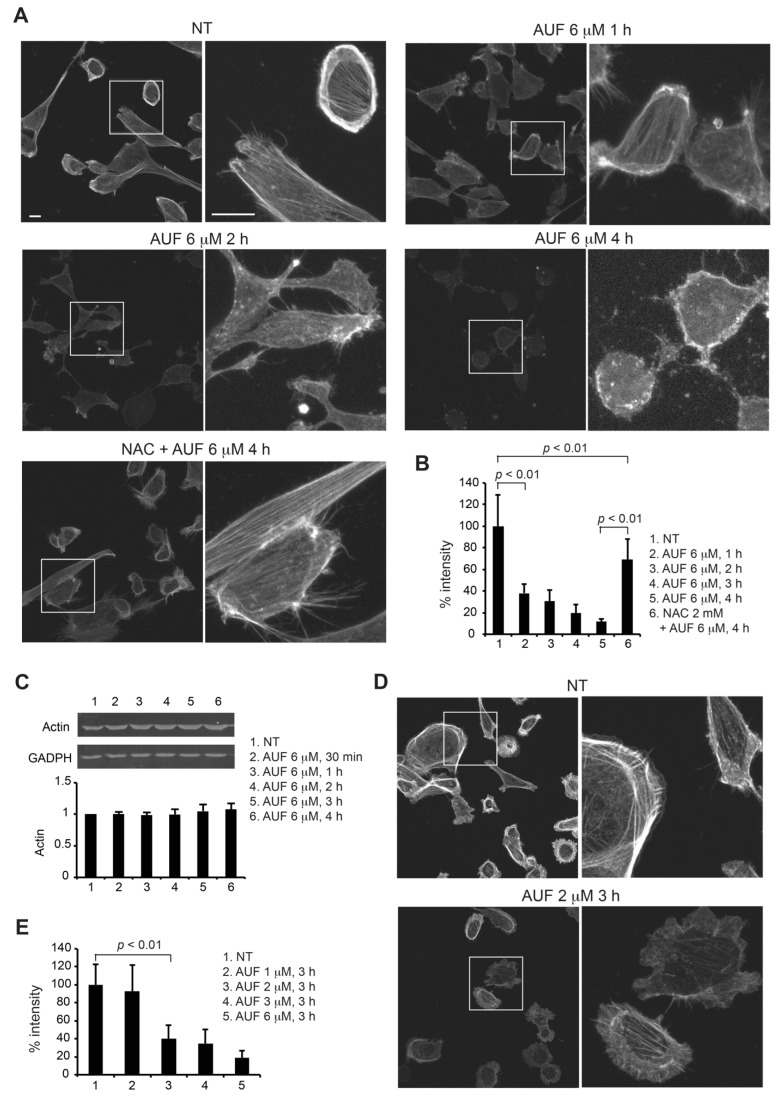
Effect of AUF on actin cytoskeleton structure. (**A**) Non-treated (NT) and 6 µM AUF-treated MDA-MB-231 cells were stained with rhodamine–phalloidin to visualize the actin cytoskeleton. Z-stack images were obtained using a Leica SP8 upright confocal microscope and merged using ImageJ software. Representative images of NT and cells treated for 1, 2, and 4 h alone or in the presence of 2 mM NAC (4 h) are presented. Adjustments were applied only in the zoom images of cell treated by AUF for 4 h to better visualize actin cytoskeleton modifications. Scale bars = 10 µm. (**B**) Change in F-actin staining intensity was measured as a percentage of NT control (set as 100%). Data points represent the measurement of at least 30 cells with the same image acquisition conditions. (**C**) Immunoblot of actin of MDA-MB-231 cells treated with indicated conditions. GADPH was used as a loading control. Protein levels quantified using ImageJ are expressed as fold change over NT control (set as 1). Original blots/gels can be found at supplementary files. (**D**) MDA-MB-231 cells, NT or treated with 1, 2, 3 and 6 μM AUF for 3 h, were stained with rhodamine–phalloidin to visualize the actin cytoskeleton. Images were obtained as (**A**). Representative images of NT and 2 μM AUF-treated cells are presented. (**E**) Change in F-actin staining intensity of the experiment described in (**D**) was measured and analyzed as in (**B**).

Proteins involved in cytoskeleton structure and function are among the most affected by AUF treatment according to the redoxome analysis (Table 1). This prompted us to investigate the state of the cell cytoskeleton in response to 6 µM AUF treatment with rhodamine-conjugated phalloidin and confocal microscopy. Non-treated MDA-MB-231 cells displayed regular, stretched and intact filamentous actin (F-actin) fibers (Figure 6A). Changes on the actin filaments were clearly visible as early as 1 h after AUF treatment, whereas F-actin fibers became shorter and irregularly destructured. After 3 to 4 h of AUF treatment, intact and regular actin filaments became rare (Figure 6A,B), which is associated with the appearance of membrane blebbing and cell shrinkage. The presence of 2 mM NAC that suppresses AUF-induced cell death preserved globally F-actin fibers, although less strongly stained by rhodamine-conjugated phalloidin (Figure 6A,B). Importantly, Western blot analysis showed the presence of similar quantities of actin in cells under above described treatment conditions (Figure 6C and Supplementary Figure S4). We then further checked the AUF dose–effect on actin filaments. Treatment with 1 µM AUF did not result in a readily detectable change in intensity of rhodamine-conjugated phalloidin staining, while a decrease in intensity of staining associated with shorter and irregularly destructured filaments was visible in the presence of 2 µM AUF for 3 h (Figure 6D,E). Consistent with the redoxome data, we revealed that AUF could dramatically and rapidly destroy cytoskeletal organization by triggering the disassembly or depolymerization of actin filaments. Since cytoskeleton serves mechanical, organizational and signaling functions within the cells, early disruption of the F-actin structure may in itself induce cell death.

## 4. Discussion

Despite the increasing interest in AUF anticancer activity, its biological mechanisms of action are still controversial. The best-known mechanism for AUF’s anticancer effect is the inhibition of TrxR activity, thus inducing the generation of ROS and cell apoptosis [4,8]. A recent unbiased chemical proteomics study confirmed TrxR1 as one of the main AUF targets [29]. While there is no doubt that AUF preferentially interacts with TrxR, whether such induced inhibition alone is sufficient to trigger cell death remains questionable. The thioredoxin system has been considered as an interesting anticancer target [30,31]. The genetic inactivation of TrxR1 or TrxR2 alone can produce a profound impact on cancer cells but often has a limited effect regarding cytotoxicity due to the presence of efficient redox backup systems [32,33]. In the current study, we found that one bolus dose of low concentrations of AUF inhibited TrxR efficiently but displayed only low cytotoxicity in MDA-MB-231 cells. Low concentrations of AUF only transiently inhibit TrxRs as suggested by the transient inhibition of BrdU incorporation (Figure 5A). Indeed, cells treated with low concentrations of AUF trigger an adaptative response by increasing the biosynthesis of reduced and active TrxR1 and other proteins [34]. It is also possible that low concentrations of AUF do not sufficiently affect other targets that contribute to AUF-induced cytotoxicity, while a cytotoxic concentration of AUF not only sustainably inhibits TrxRs but also affects other targets/mechanisms such as glutathione pathway [20], proteasome system [10,11], endoplasmic reticulum stress [35], DNA replication (see discussion below) etc., leading to massive oxidative stress and rapid cell death. The presence of NAC, a precursor of glutathione that can largely restore cellular viability in AUF-treated cells, did not restore TrxR activity. An elevation in intracellular glutathione levels following NAC treatment may prevent the inactivation of thiol groups of important target proteins, preventing cell death.

MS-based cysteine redox proteomics has been employed to analyze the effect and mechanisms of AUF. Using human colorectal adenocarcinoma HT-29 cells treated with 20 µM AUF for 2 h, Go et al. reported that the associated proteins of identified oxidized peptides primarily mapped to glycolysis, cytoskeleton remodeling, translation and cell adhesion [36]. Saeia et al. using human colorectal carcinoma HCT116 cells treated with 3 µM AUF for 2 h found that significantly oxidized peptides mapped best to the KEGG (Kyoto Encyclopedia of Genes and Genomes) pathways such as ribosome, spliceosome, glycolysis/gluconeogenesis, metabolic pathways, DNA replication and cell cycle. In contrast, the significantly reduced peptides mapped mostly to other pathways such as focal adhesion, adherens junction and ribonucleoprotein complex [29]. Using other chemical proteomics tools, the authors confirmed TrxR1 as one of the main AUF targets. Most recently, Chiappetta et al. simultaneously monitored the protein expression profiles and the cysteine oxidations in the A2780 ovarian cancer cells exposed to 0.7 μM AUF for 24 h [34]. For the oxidized Cys, mitochondrial oxidative phosphorylation and the response to inorganic substances were the most affected biological functions, while the ribosome was the most affected KEGG pathway. Their protein expression data indicated an upregulation of drug adaptation/resistance mechanisms.

We applied the MS-based redox proteomics approach in MDA-MB-231 cells treated with cytotoxic 6 µM AUF for 30 min in order to reveal unbiasedly early events that could link to AUF cytotoxicity. The very short AUF treatment time suggests that changes in protein abundance are negligible and unlikely to affect conclusions. Indeed, Western blots estimating the abundance of several randomly selected individual proteins prior to and post-AUF treatments under these treatment conditions indicate no changes in protein abundance. Redoxome and bioinformatics analysis indicates that the cysteines of proteins implicated in cell proliferation, cell division, cell cycle and cytoskeleton-associated proteins are among preferential targets of AUF treatment. These data are partially consistent with those reported by Go et al. [36] and Saeia et al. [29], also using relatively high doses of AUF and short treatment.

Based on our redoxome data, a BrdU incorporation experiment further points to the dose-dependent AUF-induced inhibition of DNA replication as an early event that could underline the anticancer effect of AUF. How does AUF treatment lead to the arrest of DNA synthesis? The most likely hypothesis is via the impact of AUF on RNR. RNR is involved in the de novo synthesis of the dNTPs. The availability of each of the four dNTPs is a limiting factor to nascent DNA strands elongation. RNR activity depends on thioredoxins and glutaredoxins as the electron donors [37]. Thioredoxins and glutaredoxins ultimately obtain the electrons from NADPH via TrxR or via glutathione reductase and glutathione. An NADPH-independent methionine-consuming pathway can also support RNR [38]. AUF is an inhibitor of TrxR and affects the glutathione pathway. Consistently, the dTTP and dGTP levels experienced rapid decreases in AUF dose- and treatment time- dependent ways. The depletion of the dNTP pool induced by lower concentrations of AUF is likely transient or partial, as BrdU incorporation partially resumed 6 h post-AUF treatment at 1, 2 or even 3 µM. Indeed, RNR1, RNR2 and TrxR1 were found to be upregulated in A2780 ovarian cancer cells exposed to 0.7 μM AUF for 24 h, which is a concentration corresponding to the 72 h exposure IC50 dose [34]. Upon exposure to 6 µM AUF, BrdU incorporation was totally inhibited in a durable manner. Such a cytotoxic concentration may inhibit strongly and persistently TrxR and other backup pathways that support RNR, leading to persistent DNA replication fork stalling and replication stress. These mechanisms may contribute to AUF-induced cell death [39,40].

Cysteine residues in cytoskeleton-associated proteins were another preferential target of AUF as suggested by our redoxome and bioinformatics analysis. The cytoskeletal molecules can be classified into three main classes, namely the actin filaments, microtubules and intermediate filaments. Actin itself is the most redox-sensitive and the most dynamic of the three cytoskeletal elements [41]. The reversible polymerization and depolymerization of actin allow the cytoskeleton to be dynamic in response to different conditions, such as for cell migration, attachment, division, and polarization. Numerous cytoskeletal-associated proteins also help to regulate the spatial and temporal distribution of the cytoskeleton. Actin filaments underwent rapid and visible modifications in response to moderate or cytotoxic AUF concentrations, while actin levels remained constant, suggesting a drastic effect of AUF on depolymerization of actin filaments. Indeed, the oxidation of actin, actin-binding proteins, and/or proteins in signaling cascades that regulate the actin cytoskeleton has profound consequences on cells, affecting cell adhesion and migration, cell contraction, cell division and proliferation and cell death [42]. In addition, the cytoskeleton and its associated molecules have been considered as therapeutic targets [41]. However, the oxidation of specific proteins involved in these critical cellular functions and its functional consequences remain largely unknown. Actin cytoskeleton could be a key target of AUF, leading to cell death. Whether other cytoskeleton components are affected, how AUF induces rapid disruption of the cytoskeletal structure, how the oxidation of cytoskeletal proteins identified in redoxome analysis affects cytoskeletal structure and function, and finally, how AUF-induced cytoskeletal disruption links to cell death are questions that need to be addressed.

Drug experimental investigation should be, when possible, in line with a pharmacological dose relevant for clinical uses. AUF has been in clinical use for the treatment of rheumatoid arthritis with a known toxicity profile [43]. A more recent Phase I study also showed that when AUF was taken orally at 6 mg/day, the mean gold maximum concentration in plasma at day 7 reached 1.58 µM [44]. This recommended dose for rheumatoid arthritis was generally well tolerated. The concentrations of AUF used in laboratory studies vary very much. It is not always easy to apply known “pharmacological doses” in the laboratory when performing the experiments in different cell models with a bolus dose of AUF. For example, Go et al. treated colorectal carcinoma cells HT-29 with 20 µM AUF for 2 h to analyze the effect of AUF with cysteine redox proteomics approach [36], while Saei et al. reported their redox proteomics study using 3 µM AUF and HCT116 colorectal carcinoma cells [29]. A transcriptomics study was carried out with nine cell lines exposed to AUF at 10 µM for 6 h [45]. These investigations using different cell models, various AUF concentrations and treatment times generate a yet incomplete but valuable vision of its mechanisms of action.

## 5. Conclusions

The mechanisms underlying anticancer activity of AUF appear to be complex and at least partially cancer cell type- or dose-dependent. We conducted the present study with particular attention to identify the early events leading to AUF-induced cytotoxicity using moderate and cytotoxic concentrations of AUF. We evidenced that AUF-mediated TrxR inhibition alone may not be sufficient to induce efficiently cell death. Cytotoxic doses of AUF elicited rapid and drastic intracellular oxidative stress affecting mitochondria, cytoplasm and nucleus. Based on the indications from redox proteome data, we showed experimentally that AUF treatment triggered a dose-dependent S-phase arrest, dTTP and dGTP depletion and disintegration of actin cytoskeleton structure. This spectrum of AUF-induced early effects should provide novel insights into the anticancer mechanisms of this promising redox molecule. Our study using TNBC cell line models suggests that some cancer types and subtypes would be particularly susceptible for AUF treatment. It will be interesting to identify predictive factors for sensitivity/resistance to AUF. For cancer cells that display only moderate sensitivity to AUF, a better understanding of mechanisms of action of AUF should help identify AUF-based rational drug combinations that increase global anticancer efficacy and decrease the dosage and side effects of single drugs.

## Figures and Tables

**Table 1:** Top ten biological processes involved by the 161 proteins with significant fold change (DAVID program analysis).

GO Term	Biological Process	Count	%	*p* Value
GO:0098609	cell–cell adhesion	18	11.18	6.0825 × 10^−10^
GO:0055114	oxidation–reduction process	13	8.07	0.0093
GO:0008283	cell proliferation	11	6.83	0.0023
GO:0043066	negative regulation of apoptotic process	10	6.21	0.0264
GO:0007010	cytoskeleton organization	9	5.59	1.3107 × 10^−4^
GO:0016032	viral process	9	5.59	0.0069
GO:0006457	protein folding	8	4.97	0.0015
GO:0051301	cell division	8	4.97	0.0452
GO:0007049	cell cycle	7	4.35	0.0159
GO:0007067	mitotic nuclear division	7	4.35	0.0284

## Data Availability

The data presented in this study are available on request from the corresponding author.

## Supplementary Materials

The following supporting information can be downloaded at: https://www.mdpi.com/article/10.3390/cancers14194864/s1, Figure S1: Redox state of PRDX3 as givens in main Figure 3A; Figure S2: Redox state of cyto- and nuc-HyPer sensors in MDA-MB-231 cells as given in main Figure 3B; Table S1: List of the differentially oxidized cysteine-containing proteins in MDA-MB-231 cells upon 6 μM AUF treatment for 30 min; Figure S3: Effect of AUF on cell cycle; Figure S4: Immunoblot of actin of MDA-MB-231 cells treated with indicated conditions as given in main Figure 6C.
