# Supplementary material for: Novel Insights into Redox-Based Mechanisms for Auranofin-Induced Rapid Cancer Cell Death
Source: Cancers (Basel). 2022 Oct 5;14(19):4864. doi: 10.3390/cancers14194864 (PMC9562029; doi:10.3390/cancers14194864)
Supplement: Supplementary file 1 [file cancers-14-04864-s001.zip › Figure S2.pdf]

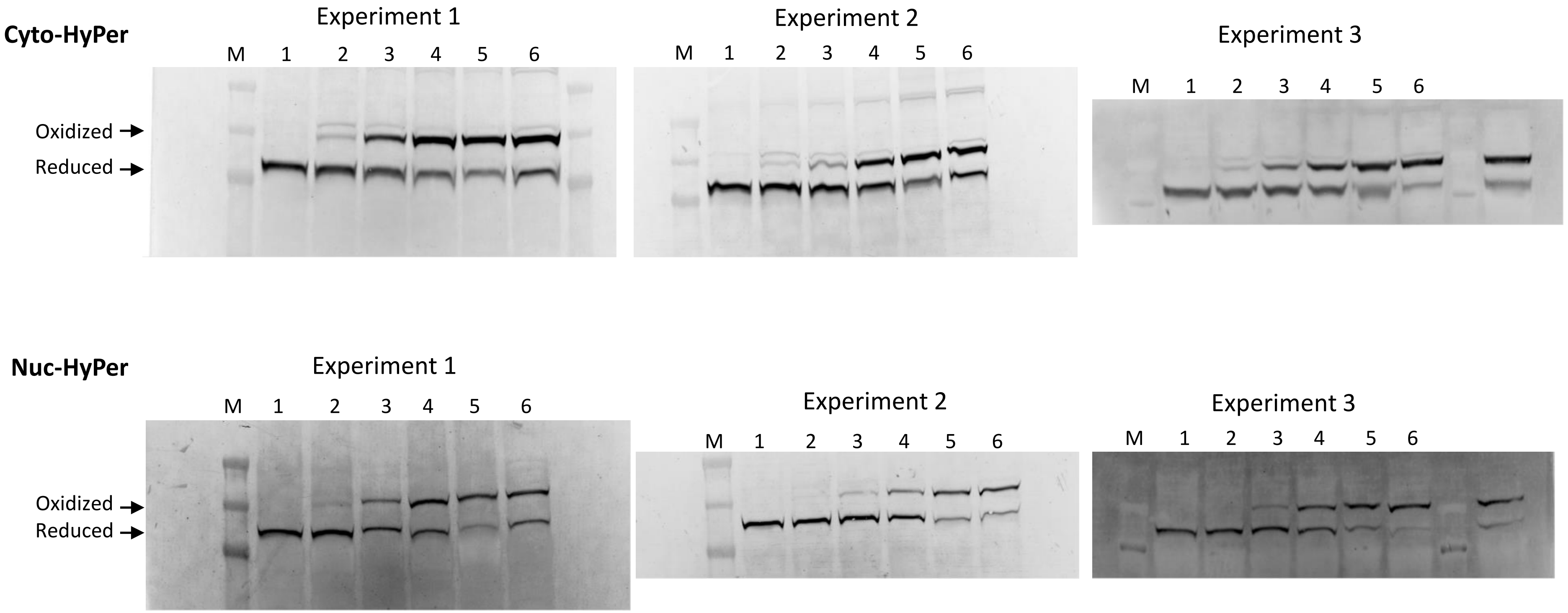

Figure S2. Redox state of cyto- and nuc-HyPer sensors in MDA-MB-231 cells as given in main Figure 3B. Original blots/gels of three experiments are presented. M: Marker; 1. NT (non-treated); 2. AUF 6 µM, 15 min; 3. AUF 6 µM, 30 min; 4. AUF 6 µM, 1 h; 5. AUF 6 µM, 4 h; 6. H2O2 positive control.
