# Supplementary material for: Novel Insights into Redox-Based Mechanisms for Auranofin-Induced Rapid Cancer Cell Death
Source: Cancers (Basel). 2022 Oct 5;14(19):4864. doi: 10.3390/cancers14194864 (PMC9562029; doi:10.3390/cancers14194864)
Supplement: Supplementary file 1 [file cancers-14-04864-s001.zip › Figure S3.pdf]

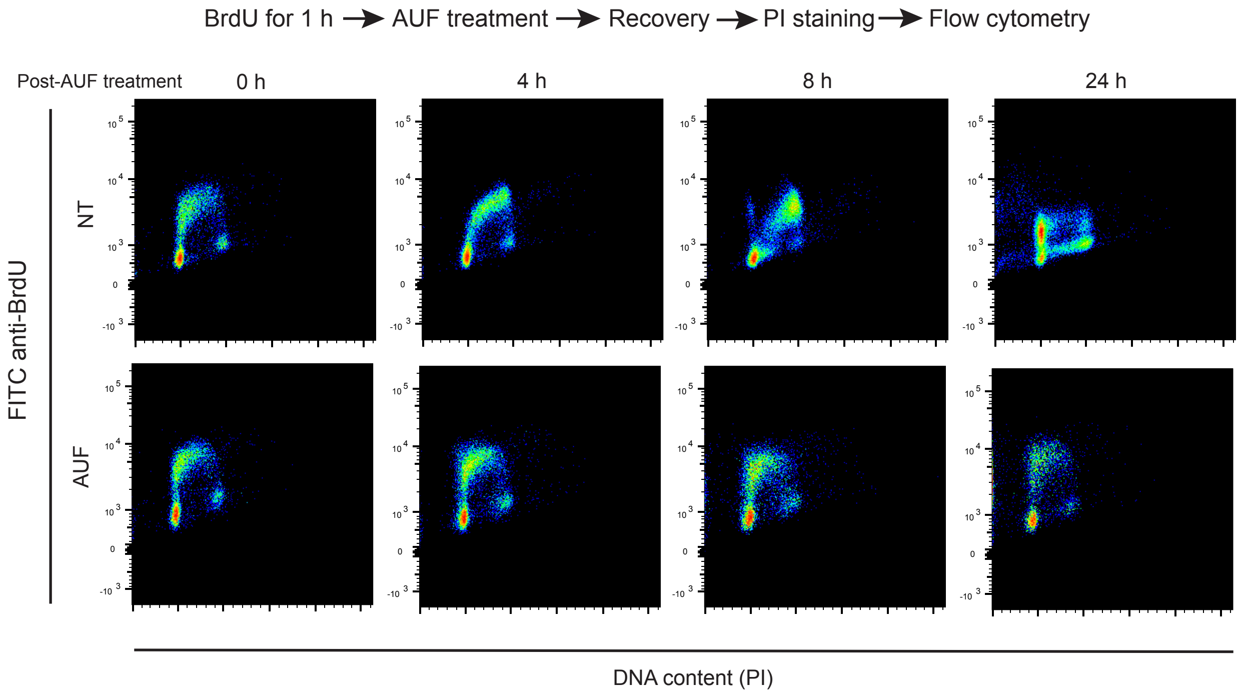

Figure S3. Effect of AUF on cell cycle. MDA-MB-231 cells were labeled with BrdU for 1 h and then subjected to two conditions for 30 min: non-treated (NT) or treated with 6 μM AUF. After a defined recovery time in growth media (0, 4, 8, or 24 h), cells were removed and proceeded for PI staining and flow cytometry. Representative graphs of two experiments are shown.
