# Supplementary material for: Novel Insights into Redox-Based Mechanisms for Auranofin-Induced Rapid Cancer Cell Death
Source: Cancers (Basel). 2022 Oct 5;14(19):4864. doi: 10.3390/cancers14194864 (PMC9562029; doi:10.3390/cancers14194864)
Supplement: Supplementary file 1 [file cancers-14-04864-s001.zip › Figure S4.pdf]

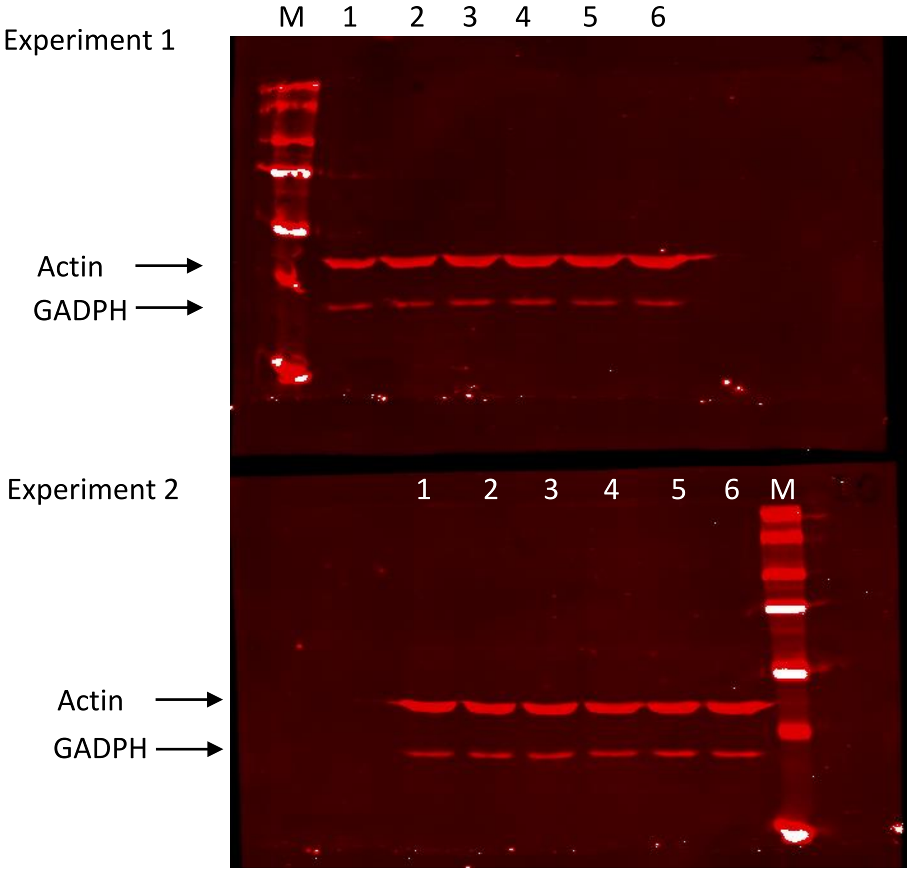

Figure S4. Immunoblot of actin of MDA-MB-231 cells treated with indicated conditions as given in main Figure 6C. Original blots/gels of two experiments are presented. M: Marker**;** 1. NT (non-treated); 2. AUF 6 µM, 30 min; 3. AUF 6 µM, 1 h; 4. AUF 6 µM, 2 h; 5. AUF 6 µM, 3 h; 6. AUF 6 µM, 4 h.
