# Supplementary material for: Novel Insights into Redox-Based Mechanisms for Auranofin-Induced Rapid Cancer Cell Death
Source: Cancers (Basel). 2022 Oct 5;14(19):4864. doi: 10.3390/cancers14194864 (PMC9562029; doi:10.3390/cancers14194864)
Supplement: Supplementary file 1 [file cancers-14-04864-s001.zip › Table S1.pdf]

Table S1. List of the differentially oxidized cysteine-containing proteins in MDA-MB-231 cells upon 6 μM AUF treatment for 30 min

161 proteins accession	Anova (*p* value)		Fold change (ratio)*		Highest mean condition	Lowest mean condition	Gene
	Exp1	Exp2	Exp1	Exp2			
Q9UMR2	2.03E-05	8.54E-04	**2.327**	**2.432**	AUF	NT	***DDX19B***
Q5BKZ1	2.30E-05	3.87E-04	**2.302**	**1.797**	AUF	NT	***ZNF326***
Q9NSB4	1.28E-04	1.76E-02	**8.168**	**40.143**	AUF	NT	***EIF2B3***
Q8NBJ7	1.80E-04	1.41E-02	**-1.679**	**-1.318**	NT	AUF	***SUMF2***
P51858	2.28E-04	1.23E-03	**-13.415**	**-5.979**	NT	AUF	***HDGF***
Q15436	2.46E-04	2.25E-02	**1.524**	**1.455**	AUF	NT	***SEC23A***
Q9NU22	2.50E-04	6.13E-05	**3.525**	**2.363**	AUF	NT	***MDN1***
P31151	2.81E-04	1.24E-02	**1.978**	**4.300**	AUF	NT	***S100A7***
P50453	4.25E-04	3.38E-03	**3.407**	**1.592**	AUF	NT	***SERPINB9***
O60610	4.58E-04	1.21E-02	**-4.444**	**-1.755**	NT	AUF	***DIAPH1***
P13489	6.33E-04	6.98E-03	**2.934**	**1.778**	AUF	NT	***RNH1***
Q14566	6.44E-04	1.16E-03	**1.607**	**1.555**	AUF	NT	***MCM6***
P17980	7.56E-04	1.36E-02	**2.329**	**1.533**	AUF	NT	***PSMC3***
P10599	7.56E-04	9.71E-03	**2.732**	**1.843**	AUF	NT	***TXN***
Q14847	7.99E-04	2.09E-03	**2.267**	**1.898**	AUF	NT	***LASP1***
P05455	7.99E-04	2.05E-02	**-2.399**	**-1.567**	NT	AUF	***SSB***
Q00610	8.31E-04	1.88E-03	**2.149**	**2.309**	AUF	NT	***CLTC***
Q15019	8.35E-04	1.10E-02	**4.045**	**1.888**	AUF	NT	***SEPT2***
P06748	8.43E-04	2.07E-02	**-1.826**	**-2.106**	NT	AUF	***NPM1***
O00299	8.74E-04	4.13E-03	**1.695**	**2.071**	AUF	NT	***CLIC1***
Q96DI7	8.96E-04	4.46E-03	**2.352**	**1.816**	AUF	NT	***SNRNP40***
P07355	9.58E-04	2.60E-03	**1.720**	**1.543**	AUF	NT	***ANXA2***
P07477	1.22E-03	3.30E-04	**3.227**	**2.684**	AUF	NT	***PRSS1***
P46782	1.23E-03	9.73E-03	**1.951**	**1.596**	AUF	NT	***RPS5***
Q9NX58	1.46E-03	3.44E-03	**2.074**	**1.940**	AUF	NT	***LYAR***
P46063	1.53E-03	2.15E-03	**2.171**	**1.373**	AUF	NT	***RECQL***
Q13177	1.54E-03	2.02E-03	**3.374**	**2.654**	AUF	NT	***PAK2***
P50454	1.68E-03	1.86E-03	**-1.515**	**-2.845**	NT	AUF	***SERPINH1***
P25774	1.71E-03	4.75E-02	**1.921**	**1.592**	AUF	NT	***CTSS***
P61160	1.80E-03	1.55E-03	**1.847**	**1.404**	AUF	NT	***ACTR2***
P23284	1.84E-03	2.82E-02	**1.917**	**1.795**	AUF	NT	***PPIB***
Q92747	1.88E-03	2.48E-02	**2.403**	**1.735**	AUF	NT	***ARPC1A***
P60510	1.99E-03	1.55E-02	**1.886**	**1.262**	AUF	NT	***PPP4C***
Q99447	2.03E-03	1.74E-02	**1.799**	**1.302**	AUF	NT	***PCYT2***
P30479	2.17E-03	3.97E-03	**-1.289**	**-1.620**	NT	AUF	***HLA-B***
P13646	2.21E-03	5.42E-05	**2.330**	**6.467**	AUF	NT	***KRT13***
P78417	2.27E-03	3.83E-03	**1.750**	**1.777**	AUF	NT	***GSTO1***
Q9UBQ5	2.54E-03	5.93E-03	**1.618**	**1.489**	AUF	NT	***EIF3K***
Q6XZF7	2.65E-03	1.13E-03	**2.184**	**2.139**	AUF	NT	***DNMBP***
P51149	2.66E-03	2.96E-02	**1.750**	**1.528**	AUF	NT	***RAB7A***
P54577	2.72E-03	3.89E-02	**1.586**	**1.448**	AUF	NT	***YARS***
P62979	2.73E-03	5.54E-03	**2.073**	**2.120**	AUF	NT	***RPS27A***
P14314	2.74E-03	4.98E-02	**-1.925**	**-1.279**	NT	AUF	***PRKCSH***
O94804	2.84E-03	6.31E-04	**-5.098**	**-2.380**	NT	AUF	***STK10***
Q01970	3.02E-03	2.32E-02	**-2.078**	**-1.415**	NT	AUF	***PLCB3***
P23526	3.09E-03	3.74E-02	**1.725**	**1.482**	AUF	NT	***AHCY***
O43681	3.26E-03	4.64E-03	**2.266**	**1.816**	AUF	NT	***ASNA1***
P14373	3.41E-03	2.23E-02	**1.874**	**1.851**	AUF	NT	***TRIM27***

O76031	3.41E-03	2.48E-05	**1.759**	**1.759**	AUF	NT	***CLPX***
Q99575	3.59E-03	1.24E-02	**-1.386**	**-2.050**	NT	AUF	***POP1***
P56537	3.59E-03	2.20E-02	**1.794**	**1.474**	AUF	NT	***EIF6***
Q16181	3.59E-03	6.08E-03	**1.629**	**1.569**	AUF	NT	***SEPT7***
Q9Y5B8	3.79E-03	1.13E-02	**2.716**	**1.600**	AUF	NT	***NME7***
Q5THJ4	3.84E-03	5.79E-04	**5.071**	**3.172**	AUF	NT	***VPS13D***
O96013	4.13E-03	7.36E-03	**-1.836**	**-2.052**	NT	AUF	***PAK4***
Q96HE7	4.43E-03	8.03E-04	**-1.756**	**-1.706**	NT	AUF	***ERO1L***
Q12849	4.44E-03	2.62E-03	**2.046**	**1.940**	AUF	NT	***GRSF1***
Q9NUQ8	4.46E-03	3.03E-02	**1.910**	**1.577**	AUF	NT	***ABCF3***
O14929	4.51E-03	1.45E-02	**2.909**	**2.186**	AUF	NT	***HAT1***
Q96FS4	4.71E-03	9.07E-03	**-1.958**	**-2.131**	NT	AUF	***SIPA1***
Q10713	4.74E-03	2.72E-02	**2.098**	**1.330**	AUF	NT	***PMPCA***
Q12899	4.90E-03	1.20E-02	**4.545**	**1.988**	AUF	NT	***TRIM26***
O15231	5.06E-03	2.39E-03	**1.792**	**1.570**	AUF	NT	***ZNF185***
Q9UBE0	5.10E-03	3.13E-02	**1.629**	**1.335**	AUF	NT	***SAE1***
Q13148	5.19E-03	5.56E-04	**1.815**	**1.708**	AUF	NT	***TARDBP***
Q96CV9	5.30E-03	2.66E-02	**2.427**	**1.529**	AUF	NT	***OPTN***
P62805	5.31E-03	4.40E-03	**2.019**	**2.287**	AUF	NT	***HISTIH4A***
Q14527	5.42E-03	5.00E-04	**-3.178**	**-6.397**	NT	AUF	***HLTF***
P21266	5.47E-03	3.89E-02	**3.035**	**1.436**	AUF	NT	***GSTM3***
P49368	5.58E-03	3.06E-02	**1.701**	**1.400**	AUF	NT	***CCT3***
Q96CS3	5.59E-03	1.46E-02	**-1.616**	**-1.498**	NT	AUF	***FAF2***
P06753	5.68E-03	6.34E-03	**1.570**	**1.758**	AUF	NT	***TPM3***
Q15031	6.41E-03	4.02E-03	**-1.757**	**-2.293**	NT	AUF	***LARS2***
P62857	6.56E-03	3.87E-03	**2.977**	**3.442**	AUF	NT	***RPS28***
P04637	6.64E-03	1.31E-02	**1.881**	**1.527**	AUF	NT	***TP53***
P06733	6.92E-03	9.66E-03	**1.516**	**1.625**	AUF	NT	***ENO1***
P98175	7.37E-03	1.55E-02	**1.410**	**1.629**	AUF	NT	***RBM10***
P50990	7.41E-03	2.03E-02	**2.009**	**1.353**	AUF	NT	***CCT8***
Q92615	7.79E-03	1.47E-03	**1.876**	**2.453**	AUF	NT	***LARP4B***
P46821	8.00E-03	9.53E-03	**-1.580**	**-1.488**	NT	AUF	***MAP1B***
P41214	8.29E-03	2.73E-03	**3.158**	**1.996**	AUF	NT	***EIF2D***
Q9NVC6	8.65E-03	4.75E-02	**1.559**	**1.285**	AUF	NT	***MED17***
Q9NSV4	8.67E-03	8.06E-03	**-2.292**	**-1.741**	NT	AUF	***DIAPH3***
P09104	8.76E-03	1.95E-04	**1.784**	**1.527**	AUF	NT	***ENO2***
Q08AE8	8.85E-03	1.40E-03	**1.190**	**2.225**	AUF	NT	***SPIRE1***
P62826	8.89E-03	3.29E-03	**1.599**	**1.988**	AUF	NT	***RAN***
Q16222	8.90E-03	1.63E-02	**1.878**	**1.534**	AUF	NT	***UAP1***
Q9HAU5	9.43E-03	3.52E-02	**-2.190**	**-2.791**	NT	AUF	***UPF2***
Q13509	9.59E-03	1.71E-02	**-1.464**	**-1.304**	NT	AUF	***TUBB3***
P63010	1.06E-02	2.90E-03	**-1.376**	**-1.851**	NT	AUF	***AP2B1***
Q9H2D6	1.06E-02	1.26E-03	**1.480**	**1.519**	AUF	NT	***TRIOBP***
Q06830	1.11E-02	2.82E-03	**1.320**	**1.529**	AUF	NT	***PRDX1***
P13647	1.14E-02	2.75E-03	**1.121**	**2.183**	AUF	NT	***KRT5***
Q9BWF3	1.15E-02	5.31E-04	**1.180**	**4.490**	AUF	NT	***RBM4***
P20839	1.15E-02	3.42E-02	**3.489**	**3.918**	AUF	NT	***IMPDH1***
P01040	1.24E-02	8.54E-03	**2.135**	**2.534**	AUF	NT	***CSTA***
P13674	1.24E-02	1.15E-03	**-1.740**	**-2.165**	NT	AUF	***P4HA1***
Q7Z3B3	1.29E-02	1.44E-02	**-1.926**	**-1.923**	NT	AUF	***KANSL1***
P27824	1.30E-02	9.67E-03	**-1.686**	**-1.350**	NT	AUF	***CANX***
P02788	1.43E-02	3.58E-03	**1.601**	**1.768**	AUF	NT	***LTF***
O96008	1.47E-02	8.49E-04	**1.480**	**1.516**	AUF	NT	***TOMM40***
P20339	1.50E-02	1.54E-03	**2.633**	**2.028**	AUF	NT	***RAB5A***
P12429	1.52E-02	2.21E-02	**2.103**	**1.425**	AUF	NT	***ANXA3***
Q9NVA2	1.57E-02	1.15E-02	**1.545**	**1.380**	AUF	NT	***SEPT11***
P29401	1.61E-02	1.33E-02	**1.603**	**1.443**	AUF	NT	***TKT***

Q99496	1.63E-02	1.98E-02	**1.990**	**1.923**	AUF	NT	***RNF2***
P62913	1.63E-02	2.99E-03	**1.784**	**1.660**	AUF	NT	***RPL11***
Q9Y6K9	1.70E-02	3.17E-02	**2.106**	**1.654**	AUF	NT	***IKBKG***
P22061	1.74E-02	3.43E-03	**1.686**	**2.136**	AUF	NT	***PCMT1***
Q9UK59	1.77E-02	3.34E-02	**2.165**	**2.381**	AUF	NT	***DBR1***
P84022	1.78E-02	2.60E-03	**-3.095**	**-3.163**	NT	AUF	***SMAD3***
Q9Y2K7	1.81E-02	2.83E-03	**1.666**	**1.783**	AUF	NT	***KDM2A***
Q9ULD4	1.82E-02	9.71E-03	**-1.682**	**-2.327**	NT	AUF	***BRPF3***
Q9BTX1	1.83E-02	4.42E-03	**-1.703**	**-1.849**	NT	AUF	***NDC1***
P27816	1.85E-02	5.04E-03	**-1.629**	**-1.590**	NT	AUF	***MAP4***
Q9ULC5	1.87E-02	3.15E-03	**1.617**	**1.415**	AUF	NT	***ACSL5***
P28072	1.87E-02	5.40E-03	**1.636**	**2.021**	AUF	NT	***PSMB6***
P23919	1.96E-02	2.77E-02	**1.812**	**1.630**	AUF	NT	***DTYMK***
P00338	2.03E-02	2.94E-03	**1.538**	**1.630**	AUF	NT	***LDHA***
Q14202	2.06E-02	2.23E-02	**-2.560**	**-1.560**	NT	AUF	***ZMYM3***
P02462	2.13E-02	1.38E-03	**14.698**	**4.115**	AUF	NT	***COL4A1***
O15269	2.24E-02	8.27E-03	**1.852**	**1.590**	AUF	NT	***SPTLC1***
P49711	2.37E-02	1.46E-02	**-1.859**	**-2.128**	NT	AUF	***CTCF***
P16152	2.37E-02	2.42E-02	**1.553**	**1.823**	AUF	NT	***CBR1***
P17301	2.43E-02	3.72E-02	**-1.334**	**-1.540**	NT	AUF	***ITGA2***
Q15323	2.58E-02	4.10E-04	**1.832**	**5.191**	AUF	NT	***KRT31***
P51532	2.62E-02	1.65E-02	**1.559**	**1.567**	AUF	NT	***SMARCA4***
Q99729	2.64E-02	3.11E-02	**-1.411**	**-1.691**	NT	AUF	***HNRNPAB***
O60701	2.65E-02	7.60E-03	**1.713**	**1.414**	AUF	NT	***UGDH***
O94851	2.69E-02	4.98E-02	**-1.690**	**-1.317**	NT	AUF	***MICAL2***
Q13586	2.72E-02	3.92E-02	**-1.754**	**-1.263**	NT	AUF	***STIMI***
Q96RS6	2.78E-02	2.59E-02	**1.292**	**1.512**	AUF	NT	***NUDCD1***
P63104	2.87E-02	2.35E-02	**1.402**	**1.671**	AUF	NT	***YWHAZ***
P48637	2.89E-02	2.31E-02	**2.524**	**2.047**	AUF	NT	***GSS***
Q9BVK6	3.02E-02	1.83E-03	**-1.591**	**-2.811**	NT	AUF	***TMED9***
Q9UDY2	3.02E-02	2.00E-02	**-1.497**	**-1.614**	NT	AUF	***TJP2***
P78386	3.06E-02	7.24E-04	**3.771**	**8.792**	AUF	NT	***KRT85***
P04083	3.10E-02	2.40E-02	**2.263**	**2.294**	AUF	NT	***ANXA1***
P08133	3.14E-02	4.36E-02	**1.993**	**1.401**	AUF	NT	***ANXA6***
O14818	3.18E-02	3.37E-02	**1.709**	**1.563**	AUF	NT	***PSMA7***
P68036	3.19E-02	8.71E-03	**-2.220**	**-2.014**	NT	AUF	***UBE2L3***
Q9UBT2	3.29E-02	2.09E-02	**2.008**	**1.455**	AUF	NT	***UBA2***
O14763	3.40E-02	2.61E-04	**-2.155**	**-6.847**	NT	AUF	***TNFRSF10B***
Q9Y4K0	3.43E-02	2.31E-02	**-2.574**	**-4.551**	NT	AUF	***LOXL2***
P00558	3.45E-02	1.66E-02	**1.392**	**1.574**	AUF	NT	***PGK1***
P04150	3.56E-02	3.29E-02	**1.562**	**1.619**	AUF	NT	***NR3C1***
Q9BRX2	3.70E-02	1.10E-02	**1.415**	**1.789**	AUF	NT	***PELO***
Q92945	3.77E-02	7.05E-04	**1.381**	**1.800**	AUF	NT	***KHSRP***
Q8WTT2	3.79E-02	4.07E-02	**-1.671**	**-2.468**	NT	AUF	***NOC3L***
Q8TDD1	3.87E-02	2.09E-03	**-1.496**	**-2.529**	NT	AUF	***DDX54***
Q9Y3F4	3.89E-02	1.35E-02	**1.638**	**1.431**	AUF	NT	***STRAP***
Q9ULA0	3.92E-02	1.35E-02	**1.543**	**1.861**	AUF	NT	***DNPEP***
O43663	4.01E-02	2.01E-02	**-2.300**	**-1.669**	NT	AUF	***PRC1***
P02545	4.22E-02	1.42E-02	**1.578**	**1.624**	AUF	NT	***LMNA***
Q9NRG4	4.32E-02	1.73E-02	**1.928**	**1.712**	AUF	NT	***SMYD2***
Q99836	4.33E-02	2.85E-04	**4.445**	**3.573**	AUF	NT	***MYD88***
Q9BQ67	4.53E-02	8.40E-04	**-1.621**	**-2.044**	NT	AUF	***GRWD1***
Q6ZRP7	4.72E-02	2.21E-02	**-1.876**	**-2.445**	NT	AUF	***QSOX2***
P49023	4.78E-02	5.84E-04	**-3.003**	**-8.674**	NT	AUF	***PXN***
P28340	4.90E-02	1.44E-03	**-1.413**	**-2.620**	NT	AUF	***POLD1***
P05556	4.97E-02	1.02E-02	**-1.286**	**-1.967**	NT	AUF	***ITGB1***

The oxidized proteome of cysteine-containing proteins after 6 μM AUF treatment for 30 min in MDA-MB-231 cells were analyzed by mass spectrometry. 161 differentially oxidized proteins with a p value < 0.05 and a fold change (ratio) over 1.5 between non-treated (NT) and AUF-treated cells in at least one of the two biological experiments (Exp1 and Exp2) were identified. The fold change (ratio) was considered positive when the oxidized protein quantity was found higher in AUF-treated cells (highest mean condition: AUF) and negative when the oxidized protein quantity was found higher in non-treated cells (highest mean condition: NT).
